# Contrasting Effects of Natural Aging on the Quasi-Static Properties and Fatigue Life of Pultruded GFRP Composites

**DOI:** 10.3390/ma19183940

**Published:** 2026-09-16

**Authors:** Yandong Shi, Wenkai Li, Linjun Zhang, Peining Li

**Affiliations:** 1 Zhejiang Provincial Engineering Center of Integrated Manufacturing Technology and Intelligent Equipment, Hangzhou City University, Hangzhou 310015, China; shiyd@hzcu.edu.cn; 2 Key Laboratory of Composite Materials on Electrical Insulation, National Energy Administration, Jiangsu Shemar Electric Co., Ltd., Nantong 226017, Chinalpning@shenmapower.com (P.L.)

**Keywords:** pultruded GFRP, natural aging, fatigue life, quasi-static properties, interfacial damage

## Abstract

This study investigated the quasi-static properties, fatigue behavior, and surface and fracture morphologies of pultruded glass/epoxy composites subjected to 24 months of natural outdoor exposure in Guangzhou, Hangzhou, and Suihua, China. Quasi-static tests were performed at scheduled exposure intervals, whereas fatigue tests on exposed specimens were conducted after 24 months. The quasi-static moduli and strengths fluctuated without consistent monotonic degradation. At both selected tensile stress levels, all exposed groups exhibited lower mean tension–tension fatigue lives than the unexposed reference, with the Guangzhou specimens showing the lowest means. Compression–compression fatigue exhibited greater scatter and no uniform reduction across the exposure groups. SEM observations revealed surface resin loss, fiber exposure, and local fiber–matrix separation, with more pronounced surface changes in the examined Guangzhou specimens. These observations suggest that local matrix and interfacial damage may contribute to the reduced fatigue resistance. Under the investigated conditions, tension–tension fatigue life showed a more pronounced response to 24-month natural exposure than the measured quasi-static properties, supporting its use as a complementary indicator in integrity assessment of pultruded GFRP composites.

## 1. Introduction

Pultruded fiber-reinforced polymer (FRP) composites have been increasingly used in civil infrastructure, energy systems, transportation, and other engineering applications because of their high strength-to-weight and stiffness-to-weight ratios, corrosion resistance, and suitability for continuous industrial manufacturing [1,2,3]. Among the available material systems, glass fiber-reinforced polymer (GFRP) composites are particularly attractive owing to the relatively low cost of glass fibers and their favorable mechanical and chemical resistance. Nevertheless, the temperature sensitivity and combustibility of the organic polymer matrix remain important limitations of GFRP composites because exposure to elevated temperatures or fire can cause rapid deterioration in their mechanical performance and structural integrity [4]. Their long-term performance remains a major concern because the polymer matrix and fiber-matrix interface are susceptible to environmental degradation. Available durability studies have shown that environmental exposure may cause material deterioration even when structural components retain satisfactory short-term load-bearing capacity [1,3]. Reliable characterization of aging-induced degradation is therefore essential for durability assessment and the determination of appropriate property reduction factors for GFRP components.

During outdoor service, GFRP composites are subjected to simultaneous and seasonally varying temperature, moisture, solar radiation, precipitation, and other environmental factors. Moisture can penetrate the composite through the matrix, pores, microcracks, and fiber-matrix interfaces, resulting in matrix plasticization, swelling, hydrolysis, and deterioration of interfacial bonding [2,5,6,7,8]. Temperature variations may alter the viscoelastic response of the matrix and generate local stresses owing to the mismatch in thermal expansion between the fibers and matrix [9,10,11]. Ultraviolet radiation can induce chain scission, additional crosslinking, discoloration, and surface embrittlement, while repeated wet–dry or freeze–thaw cycles may further promote matrix cracking and interfacial damage [2,12,13]. Although glass fibers are relatively resistant to many environmental conditions, degradation of the surrounding matrix and interface can reduce stress-transfer efficiency and facilitate matrix cracking, fiber-matrix debonding, and local fiber exposure [7,8,14]. Because these mechanisms act concurrently under actual service conditions, natural outdoor exposure provides a realistic representation of aging under complex service environments, although the contribution of each environmental factor is difficult to isolate.

Previous natural weathering and in-service investigations have shown that the durability of GFRP composites depends strongly on exposure environment, duration, resin system, fiber architecture, manufacturing quality, and the mechanical property being evaluated. Al-Bastaki and Al-Madani [15] reported that atmospheric exposure and seawater immersion produced different changes in the mechanical properties of glass-reinforced polyester composites. Keller et al. [16] evaluated the long-term performance of a GFRP truss bridge exposed to seasonal conditions and found that tensile strength was more affected than tensile modulus. Sousa et al. [17] investigated polyester- and vinyl ester-based pultruded GFRP profiles subjected to natural weathering in an urban Mediterranean climate and observed different changes in their physical, viscoelastic, tensile, flexural, and shear properties. In contrast, relatively limited degradation was reported for GFRP bars exposed to a hot and arid environment [18], whereas exposure to humid, alkaline, saline, and other aggressive environments generally produced more pronounced reductions in residual mechanical properties [19,20,21,22]. The available results therefore demonstrate that degradation behavior identified for one exposure site or material system cannot necessarily be transferred to another [1,23]. Comparative natural exposure studies involving different climatic regions but consistent materials, exposure durations, and testing methods are still required.

Despite extensive research on the environmental durability of GFRP, most existing studies have assessed aging through residual quasi-static properties, including tensile, compressive, flexural, and shear strengths and moduli. The database compiled by Liu et al. [1] included more than 1900 experimental results, but the data were highly dispersed and did not yield uniform degradation relationships. Fiber-dominated longitudinal stiffness may remain comparatively stable when environmental damage is initially concentrated in the surface resin and fiber-matrix interface, whereas transverse, compressive, and interlaminar properties can be more sensitive but also more strongly affected by material heterogeneity and experimental scatter [1,7,14,22]. Consequently, limited changes in conventional static properties do not necessarily indicate the absence of aging-induced damage. Under cyclic loading, localized matrix cracks and weakened interfaces can progressively propagate and generate stress concentrations, potentially leading to a substantial reduction in fatigue life before a comparable loss in static strength becomes evident [2,24]. Environmental fatigue studies have shown that moisture, temperature, and aggressive media can alter fatigue strength, stiffness degradation, damage accumulation, and failure mechanisms in GFRP composites [25,26,27,28,29,30]. However, most of these studies employed water immersion, artificial seawater, hygrothermal conditioning, or other accelerated environments. Systematic investigations integrating multi-region natural exposure, static mechanical characterization, fatigue testing, and microscopic surface observations remain limited.

In this study, pultruded GFRP specimens from the same material batch were naturally exposed for 24 months in three representative climatic regions. Static mechanical tests, tension–tension fatigue tests, and scanning electron microscopy were used to investigate the effects of natural exposure on mechanical performance and surface damage. The results provide experimental evidence for understanding the different responses of static properties and fatigue life to natural aging and for improving the long-term durability assessment of pultruded GFRP composites.

## 2. Materials & Methods

### 2.1. Materials & Specimens

The material investigated in this study was a pultruded GFRP composite consisting of continuous CHANGHAI (Changzhou, China) ECR16-1200-968 glass fibers (1200 tex) and an SMCR22 (Nantong, China) thermosetting epoxy adhesive matrix. The densities of the glass fibers and cured epoxy matrix were 2.68 and 1.20 g/cm^3^, respectively. Burn-off measurements yielded mean fiber and matrix mass fractions of 79.97 wt.% and 20.03 wt.%, respectively. The void content was determined using a density-comparison method in accordance with JC/T 287-2010 [31], yielding a mean void volume fraction of 1.83%. Based on the constituent mass fractions and densities, with correction for the measured void content, the fiber and matrix volume fractions in the composite were approximately 62.98 vol.% and 35.20 vol.%, respectively. All specimens were machined from rectangular pultruded bars with an original cross-section of 54 mm × 34 mm. Material from the same production batch was used throughout the natural exposure and mechanical testing programs to minimize variations associated with raw materials and manufacturing conditions. The longitudinal direction of the pultruded bar was defined as the 0° fiber direction.

**Figure 1:**
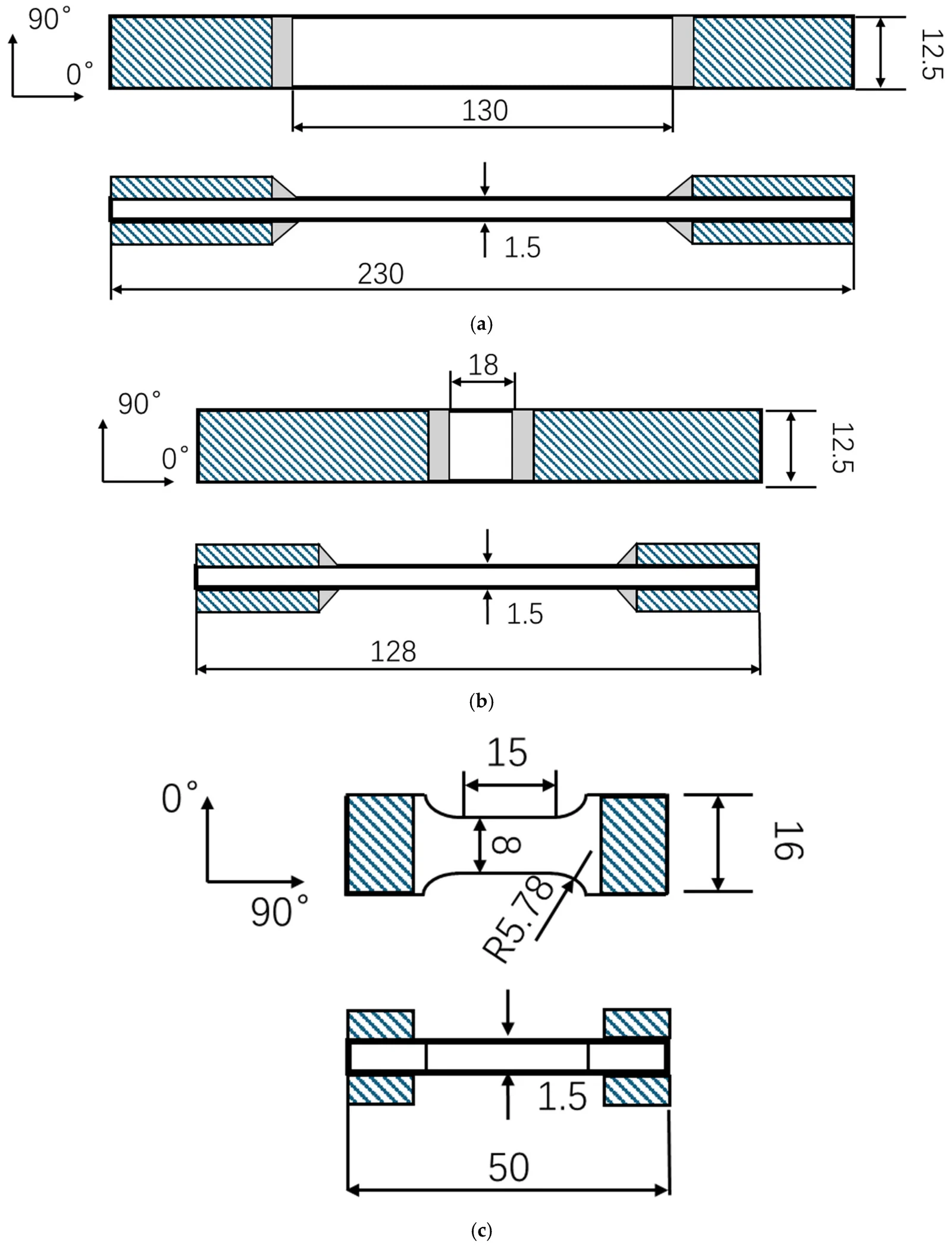
Geometries and fiber orientations of the specimens used for (**a**) longitudinal tension and tension–tension fatigue, (**b**) longitudinal compression and compression–compression fatigue, and (**c**) transverse tension. (Top and side views are shown for each specimen. All dimensions are in millimeters).

Three specimen geometries were used, as shown in Figure 1. Longitudinal tensile and tension–tension fatigue specimens were prepared according to ASTM D3039/D3039M [32], with bonded end tabs. Longitudinal compression and compression–compression fatigue specimens were prepared according to ASTM D6641/D6641M [33] and tested using a combined-loading-compression (CLC) configuration. The pultrusion process allows continuous production along the fiber direction, whereas the dimensions perpendicular to the fibers are constrained by the die cross-section. Consequently, the available transverse dimension of the bars was insufficient to machine standard transverse tensile specimens. Miniature non-standard waisted specimens were therefore used, with the fibers perpendicular to the loading direction. Transition regions were incorporated between the gripping sections and the gauge section to reduce stress concentrations near the grips and promote failure within the gauge section.

### 2.2. Natural Exposure

Natural outdoor exposure was conducted for 24 months from January 2024 to December 2025 at Guangzhou University in Guangzhou (23.05° N, 113.38° E), Hangzhou City University in Hangzhou (30.32° N, 120.15° E), and Suihua University in Suihua (46.65° N, 126.97° E), representing warm-humid subtropical, subtropical monsoon, and cold continental climates, respectively. Specimens from the same production batch were placed horizontally on iron racks approximately 1.2 m above the ground in open outdoor areas, without overhead shelter or shading of the exposed surfaces.

Specimens were not preassigned to specific retrieval intervals. Instead, they were randomly selected from the exposed specimens remaining at each site at the scheduled exposure durations. Specimens were retrieved after 3, 6, 9, 12, 15, 18, and 24 months for quasi-static testing, while specimens exposed for 24 months were retrieved for fatigue testing, as shown in Table 1. The corresponding tests commenced promptly after retrieval, without special pre-test treatment or a deliberate post-exposure conditioning period.

Table 2 presents publicly released monthly meteorological records for the three cities, obtained from the corresponding meteorological stations. These include monthly mean air temperature, monthly mean relative humidity, monthly total precipitation, and monthly sunshine duration. The records provide regional climatic context; meteorological conditions at the specimen locations were not continuously monitored. Because exposure began in January at all three sites, the shorter exposure intervals encompassed different portions of the annual seasonal cycle, which should be considered when interpreting changes with exposure duration.

### 2.3. Quasi-Static Tests

Transverse tensile, longitudinal tensile, and longitudinal compressive tests were performed to determine the corresponding elastic moduli, strengths, and stress–strain responses. Longitudinal tensile and compressive tests were conducted according to ASTM D3039/D3039M and ASTM D6641/D6641M, respectively, whereas transverse tensile tests employed the non-standard specimen described in Section 2.1. Tests were conducted under ambient laboratory conditions using an MTS servo-hydraulic testing system at a constant crosshead displacement rate of 1.4 mm/min. This rate was selected based on the authors’ established testing practice to allow sufficient data acquisition before failure, considering the relatively low failure strains of the investigated composite. The same displacement rate was used for all quasi-static tests to maintain consistency across exposure conditions. Full-field surface strains were measured using digital image correlation (DIC). During post-processing, a virtual extensometer with an initial gauge length of 10 mm was aligned with the loading direction within the specimen gauge section to determine the average axial strain. The elastic modulus was determined by linear regression of the stress–strain data over an axial strain magnitude range of 0.15–0.30%. Three specimens were tested at each exposure condition.

### 2.4. Fatigue Tests

Fatigue tests were first conducted on the unexposed material at different stress ratios, including R = −1, R = 0.1, and R = 10, to characterize its baseline fatigue response. The stress ratio was defined as R = σmin/σmax. An additional characteristic ratio of R = −0.757, corresponding to the signed ultimate compressive-to-tensile strength ratio, was considered to account for the tension–compression strength asymmetry of the material. The applied stress levels were selected as fractions of the corresponding quasi-static tensile or compressive strength and adjusted upward or downward between successive specimens to obtain fatigue data spanning approximately 10^2^–10^6^ cycles. The reference S–N curves were obtained by least-squares fitting, and the fitted relationships at different stress ratios were subsequently used to construct the constant-life Haigh diagram.

All fatigue tests were conducted under load control using an MTS servo-hydraulic testing system with a sinusoidal waveform at a frequency of 3 Hz. Testing was terminated upon specimen failure or upon reaching a prescribed cutoff of 10^6^ cycles. This cutoff was selected based on the authors’ previous testing experience to limit testing time and cost. Specimens surviving to the cutoff were recorded as runouts and were not used to fit the fatigue curve. Compression–compression fatigue tests were performed using the standard-design combined-loading-compression (CLC) fixture described in Section 2.1. The testing system was aligned before testing to minimize loading eccentricity.

For specimens naturally exposed for 24 months, fatigue tests were performed under tension–tension (R = 0.1) and compression–compression (R = 10) loading. Based on the reference S–N curves, two representative stress levels for each loading mode were selected for fatigue testing of the naturally aged specimens to reduce the required number of tests while retaining sensitivity to aging-induced fatigue degradation. The same prescribed stress levels were used across the exposure groups within each loading mode to enable direct comparison and five replicates were tested for each condition.

### 2.5. SEM Characterization

A GeminiSEM 360 scanning electron microscope (SEM; Zeiss, Oberkochen, Germany) was used to examine the surface and fracture morphologies of the GFRP specimens. All specimens were visually inspected to assess their overall surface appearance after exposure. SEM images were acquired from selected surface regions exhibiting typical features of the corresponding exposure condition. The exposed surfaces before and after natural aging were compared to identify aging-induced features, including matrix cracking, resin loss, and fiber exposure. Fracture surfaces after mechanical testing were further examined to characterize fiber breakage, matrix damage, and fiber-matrix interfacial debonding. The observations were used for qualitative morphological comparisons rather than quantitative measurements of damage area or interfacial strength.

## 3. Results

### 3.1. Evolution of Quasi-Static Properties During Natural Exposure

**Figure 2:**
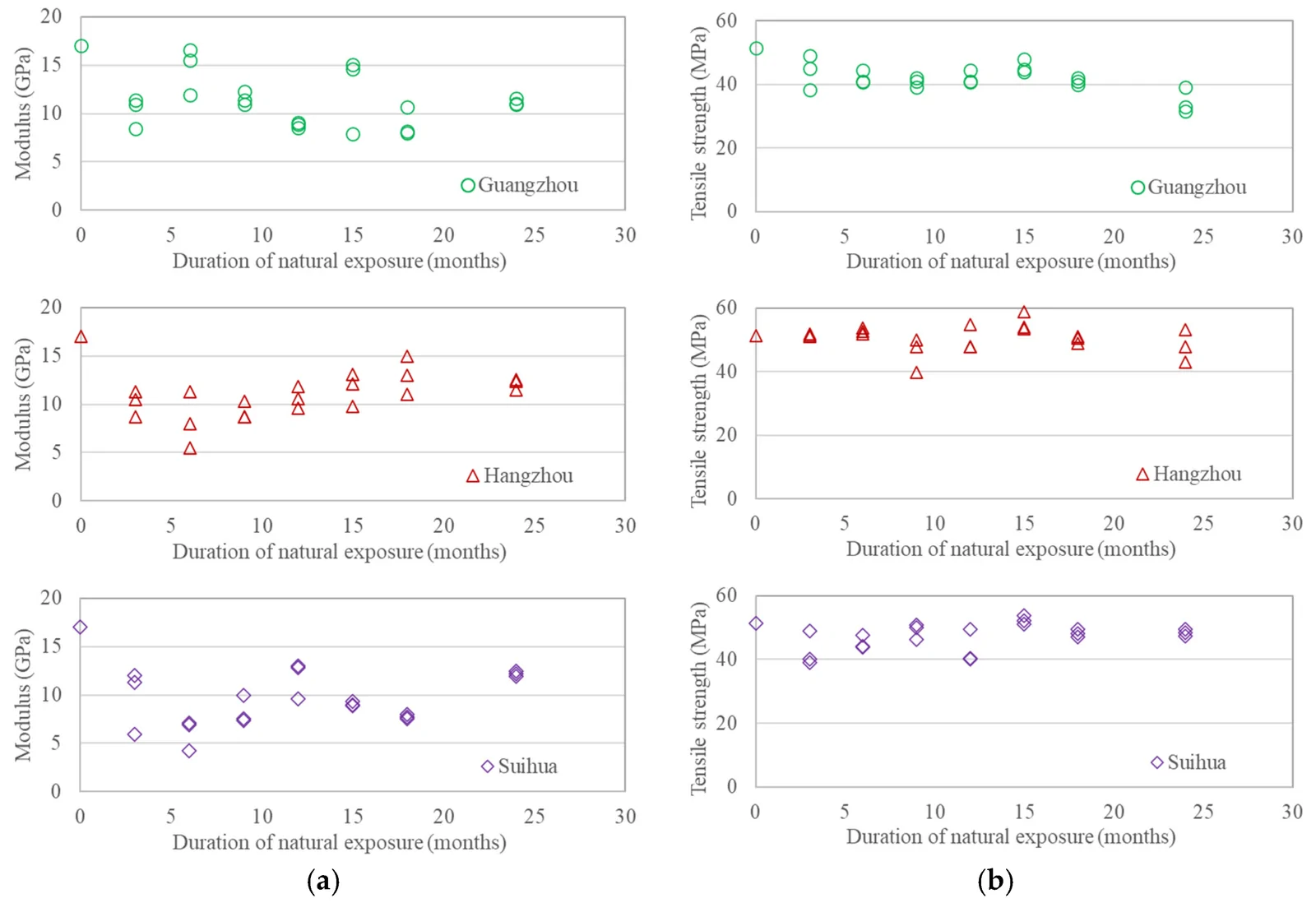
Evolution of the transverse tensile properties during natural exposure: (**a**) transverse tensile modulus and (**b**) transverse tensile strength.

**Figure 3:**
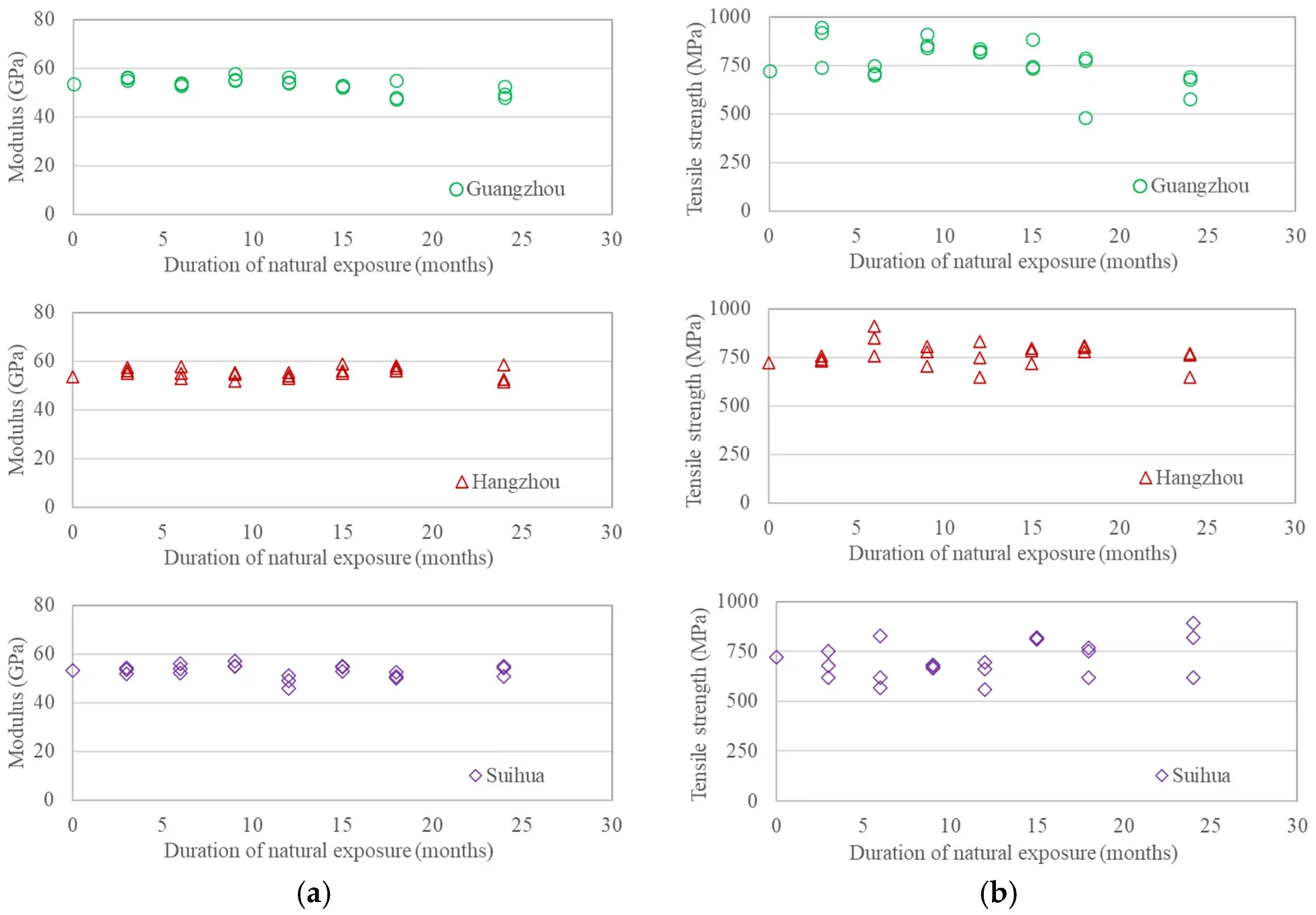
Evolution of the longitudinal tensile properties during natural exposure: (**a**) longitudinal tensile modulus and (**b**) longitudinal tensile strength.

**Figure 4:**
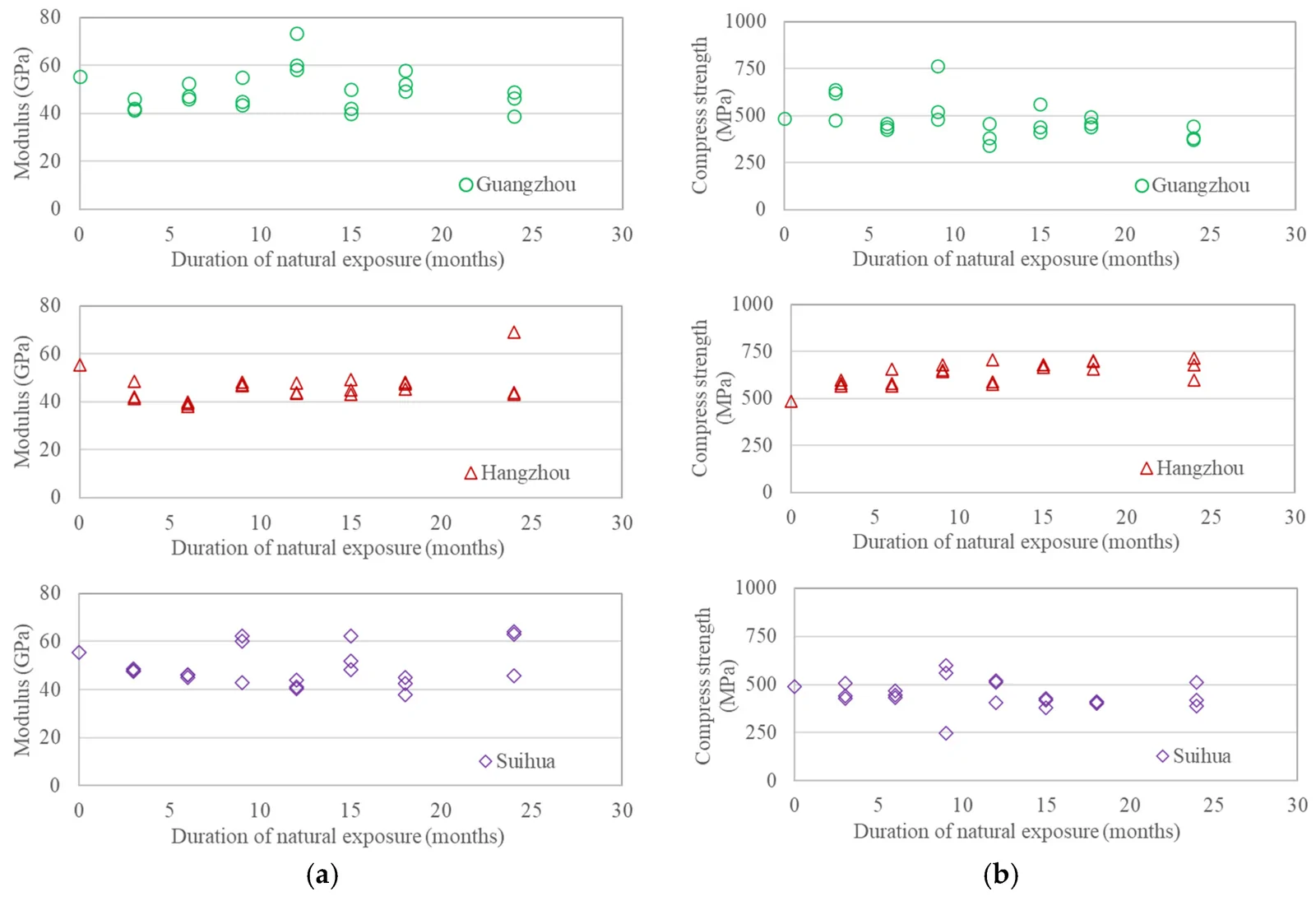
Evolution of the longitudinal compressive properties during natural exposure: (**a**) longitudinal compressive modulus and (**b**) longitudinal compressive strength.

The transverse tensile, longitudinal tensile, and longitudinal compressive properties of the GFRP specimens were measured at different stages of the 24-month natural exposure. Individual specimen measurements were shown in Figure 2, Figure 3 and Figure 4 and the corresponding means and standard deviations are summarized in Appendix A Table A1, Table A2 and Table A3. The results exhibited varying degrees of scatter depending on the loading direction and property evaluated. Overall, none of the measured quasi-static properties showed a consistent monotonic change with exposure duration in all three climatic regions.

#### 3.1.1. Transverse Tensile Properties

The transverse tensile modulus and strength measured during natural exposure are presented in Figure 2. The transverse modulus exhibited considerable specimen-to-specimen scatter at all three locations, with individual values ranging approximately from 4 to 17 GPa. No consistent monotonic relationship between the transverse modulus and exposure duration was observed. Although relatively low modulus values occurred at several intermediate exposure stages, the values measured after 24 months were approximately 11–12 GPa for all three locations.

The transverse tensile strength showed less scatter than the modulus and exhibited different temporal responses among the three locations. For the Guangzhou specimens, the strength generally decreased with exposure duration, despite some intermediate fluctuations, and reached a mean value of 34.63 MPa after 24 months. In comparison, no clear decreasing trend was observed for the Hangzhou and Suihua specimens, whose 24-month mean strengths were 48.03 and 48.36 MPa, respectively. Therefore, the three locations exhibited comparable transverse moduli at the end of the exposure period, whereas the Guangzhou specimens showed a distinctly lower transverse tensile strength. Overall, the results indicate that the transverse modulus was strongly affected by experimental scatter, while the transverse strength provided a clearer indication of the regional difference after 24 months of natural exposure.

#### 3.1.2. Longitudinal Tensile Properties

The longitudinal tensile modulus and strength measured during natural exposure are presented in Figure 3. Compared with the transverse tensile results, the longitudinal modulus exhibited relatively limited variation. Most individual values ranged from approximately 47 to 58 GPa, and no consistent monotonic relationship with exposure duration was observed at any of the three locations. After 24 months, the mean longitudinal tensile moduli were 49.95, 54.18, and 53.55 GPa for the Guangzhou, Hangzhou, and Suihua specimens, respectively.

The longitudinal tensile strength exhibited greater scatter than the modulus. For the Guangzhou specimens, relatively high strengths were measured during the early and intermediate exposure stages, followed by lower values after 18 and 24 months. The mean strength after 24 months was 649.19 MPa. The Hangzhou specimens showed no consistent deterioration with exposure duration, and their mean strength remained at 727.64 MPa after 24 months. Considerable specimen-to-specimen variation was observed for the Suihua specimens, particularly at 6, 12, and 24 months; nevertheless, their 24-month mean strength reached 777.76 MPa.

Overall, the longitudinal tensile properties did not exhibit a uniform monotonic degradation trend during the 24-month exposure period. The modulus remained comparatively stable, whereas the strength was more variable and showed different temporal responses among the three climatic regions. At the end of the exposure period, the Guangzhou specimens exhibited the lowest mean longitudinal tensile strength, while the Hangzhou and Suihua specimens retained relatively higher values.

#### 3.1.3. Longitudinal Compressive Properties

The longitudinal compressive modulus and strength measured during natural exposure are presented in Figure 4. Both properties exhibited considerable specimen-to-specimen variation, and no common monotonic trend was observed across the three exposure locations. The individual compressive modulus values ranged approximately from 38 to 73 GPa. After 24 months, the mean moduli were 44.72, 51.98, and 57.68 GPa for the Guangzhou, Hangzhou, and Suihua specimens, respectively.

The Guangzhou specimens showed relatively pronounced fluctuations in compressive strength. A high value was recorded after 9 months, followed by generally lower values during the later exposure stages. After 24 months, the mean compressive strength decreased to 398.91 MPa, representing the lowest final value among the three locations. In contrast, the compressive strength of the Hangzhou specimens generally remained above the unexposed value after 6 months, although some scatter was observed. Their mean strength after 24 months was 664.07 MPa. The Suihua specimens exhibited substantial scatter throughout the exposure period, with no consistent increasing or decreasing trend, and their 24-month mean strength was 439.20 MPa.

Overall, the longitudinal compressive results did not indicate a uniform degradation trajectory during the 24-month natural exposure. The Guangzhou specimens exhibited lower modulus and strength at the end of exposure, whereas the Hangzhou specimens retained the highest compressive strength. The pronounced scatter, particularly in the Guangzhou and Suihua groups, indicates that the compressive properties alone cannot provide a consistent measure of aging progression.

According to the above results, no consistent monotonic trends were observed in the quasi-static properties during the 24-month natural exposure period. Environmental aging may introduce localized matrix and interfacial damage before causing a readily detectable reduction in quasi-static properties. Such damage can progressively accumulate under cyclic loading and may therefore be more clearly reflected in fatigue behavior. Accordingly, the fatigue characteristics of the unexposed material and the fatigue lives of the naturally aged specimens are examined in the following sections.

### 3.2. Fatigue Behavior of the Unexposed Material

**Figure 5:**
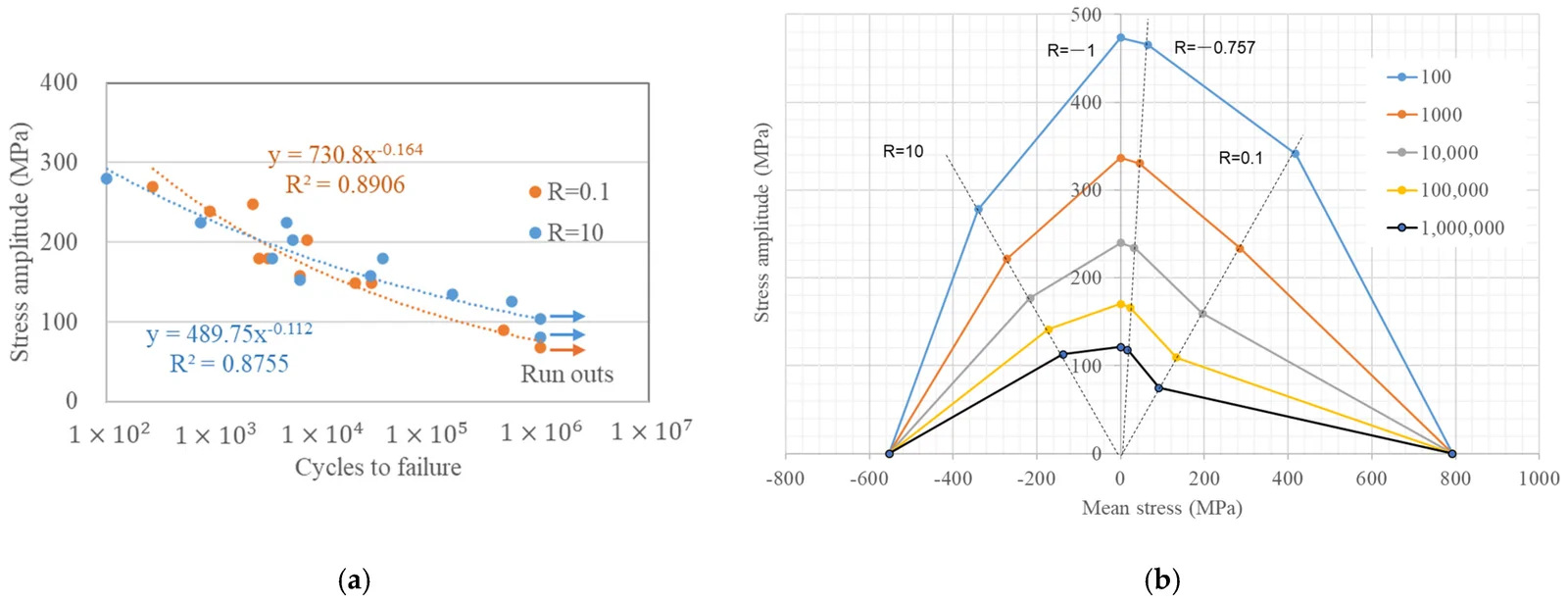
Fatigue behavior of the unexposed GFRP: (**a**) S–N relationships under tension–tension (R = 0.1) and compression–compression (R = 10) loading and (**b**) constant-life Haigh diagram showing the combined effects of stress amplitude, mean stress, and tension–compression strength asymmetry.

The fatigue behavior of the unexposed GFRP is presented in Figure 5 in terms of the S–N relationships and constant-life Haigh diagram. Figure 5a presents the S–N curves at R = 0.1 and R = 10, which were selected for display because these two stress ratios were also used in the subsequent fatigue tests on the naturally aged specimens. As shown in Figure 5a, the fatigue lives under both tension–tension (R = 0.1) and compression–compression (R = 10) loading increased with decreasing stress amplitude. Despite some scatter in the experimental data, power-law curves obtained by least-squares fitting provided a reasonable representation of the overall fatigue responses, with coefficients of determination of 0.8906 and 0.8755 for tension–tension and compression–compression loading, respectively.

The two S–N curves intersected within the investigated life range, indicating that the relative fatigue responses under tension–tension and compression–compression loading depended on the applied stress level. In the high-stress, low-cycle regime, the tension–tension curve was located above the compression–compression curve, indicating a longer tension–tension fatigue life at the same stress amplitude. As the stress amplitude decreased, the tension–tension curve declined more steeply and crossed the compression–compression curve. Consequently, in the low-stress, high-cycle regime, the compression–compression specimens exhibited a longer predicted fatigue life at the same stress amplitude. Therefore, neither loading mode consistently produced a longer fatigue life over the entire stress range. This crossover also indicates that stress amplitude alone is insufficient for comparing different loading modes because their mean stresses and maximum stress levels differ substantially.

The combined effects of stress amplitude and mean stress are illustrated by the Haigh diagram in Figure 5b. The constant-life envelopes were constructed from the fitted S–N relationships at different stress ratios by determining the stress amplitude and mean stress corresponding to each selected fatigue life. Thus, Figure 5a shows the stress–life relationships at two selected stress ratios, whereas Figure 5b presents constant-life relationships across different stress ratios. The two representations provide complementary descriptions of the baseline fatigue response. With increasing target life, the envelopes progressively contracted, showing that the estimated stress amplitude at a given fatigue life decreased over the investigated range of mean stress. The envelopes were asymmetric about the zero-mean-stress axis, reflecting the tension–compression asymmetry of the material. The ratio R = −0.757, determined from the signed ultimate compressive-to-tensile strength ratio, characterizes this tension–compression strength asymmetry.

The Haigh diagram further confirmed that the relative effects of tension-dominated and compression-dominated loading varied with fatigue life. The shapes and relative positions of the constant-life envelopes changed as the target life increased, consistent with the crossover between the tension–tension and compression–compression S–N curves. These reference results demonstrate the strong dependence of fatigue behavior on both loading mode and stress level. Accordingly, the effects of natural aging were subsequently evaluated under identical stress ratios and selected stress levels to avoid confounding the aging effect with differences in loading conditions.

### 3.3. Fatigue Life After 24 Months of Natural Exposure

**Figure 6:**
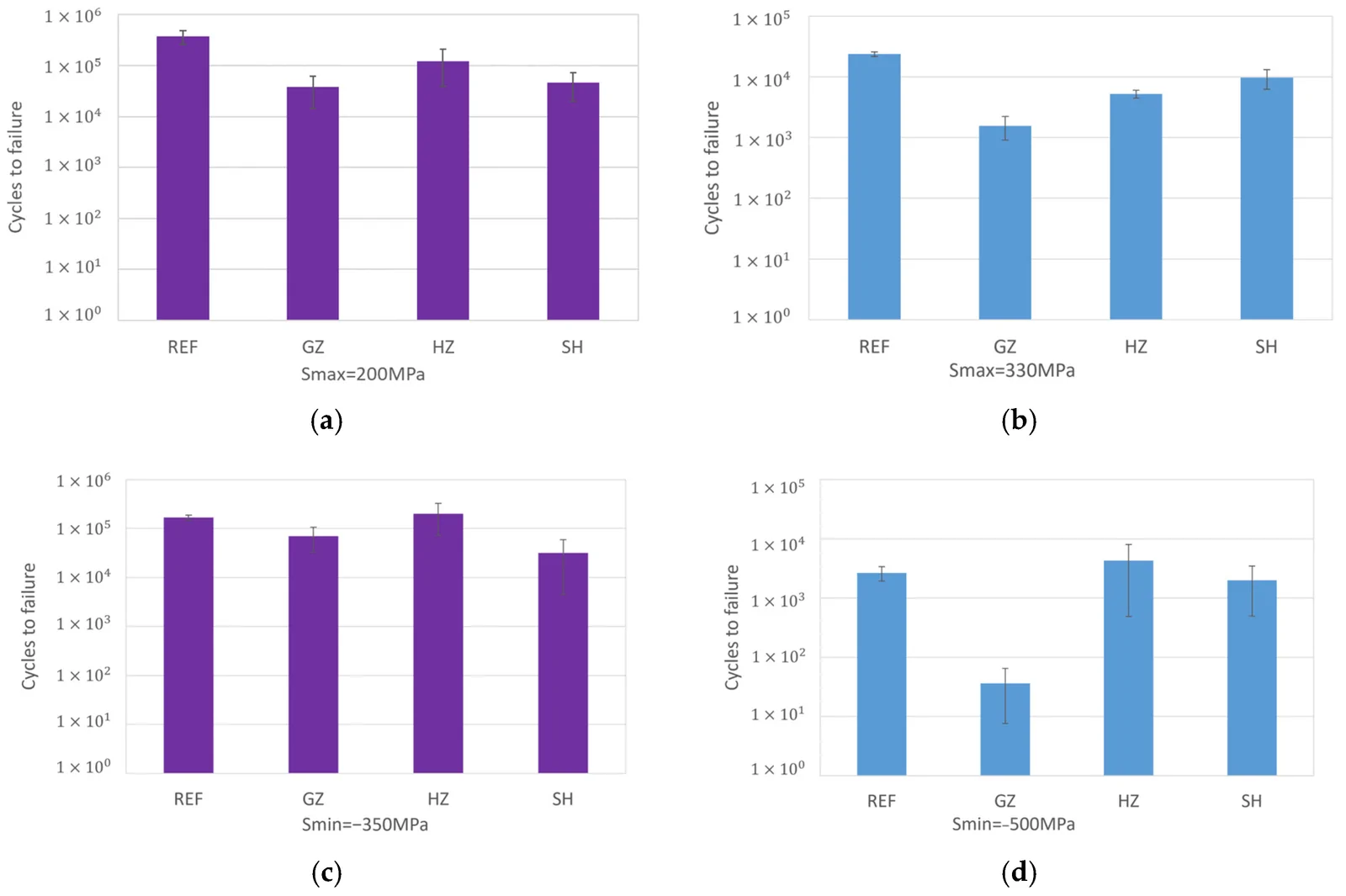
Fatigue lives of the unexposed reference material and GFRP specimens after 24 months of natural exposure: tension–tension loading at maximum stresses of (**a**) 200 MPa and (**b**) 330 MPa, and compression–compression loading at minimum stresses of (**c**) −350 MPa and (**d**) −500 MPa.

The fatigue lives of the GFRP specimens after 24 months of natural exposure are compared with those of the unexposed reference material in Figure 6. For tension–tension loading (R = 0.1), two maximum stress levels of 200 and 330 MPa were selected, corresponding to minimum stresses of 20 and 33 MPa and stress amplitudes of 90 and 148.5 MPa, respectively. For compression–compression loading (R = 10), minimum stresses of −350 and −500 MPa were applied, with corresponding maximum stresses of −35 and −50 MPa and stress amplitudes of 157.5 and 225 MPa, respectively. The two stress levels for each loading mode were selected from the reference S-N curves to represent relatively low and high stress fatigue conditions. These prescribed stress levels were kept unchanged across the exposure groups rather than being rescaled according to the residual strength after exposure; the comparisons therefore describe fatigue life under the same nominal loading conditions.

As shown in Figure 6a, at a maximum tensile stress of 200 MPa, all three naturally exposed groups exhibited shorter tension–tension fatigue lives than the unexposed reference material. The Guangzhou specimens showed the lowest mean fatigue life, while the Hangzhou specimens retained the highest fatigue life among the exposed groups. The Suihua specimens also exhibited a pronounced reduction relative to the reference material. At the higher maximum stress of 330 MPa, as shown in Figure 6b, the fatigue lives of all exposed groups were again lower than the reference value. The mean fatigue life increased in the order of Guangzhou, Hangzhou, and Suihua, with Guangzhou showing the greatest reduction. Thus, although the relative positions of Hangzhou and Suihua varied between the two stress levels, the Guangzhou specimens consistently exhibited the shortest tension–tension fatigue life.

The compression–compression results exhibited greater variability and a less consistent environmental response. At a minimum stress of −350 MPa (Figure 6c), the Guangzhou and Suihua specimens showed lower mean fatigue lives than the reference material, whereas the Hangzhou specimens exhibited a higher mean fatigue life. Among the exposed groups, Suihua showed the shortest mean fatigue life at this loading level. At the higher compressive load, with a minimum stress of −500 MPa (Figure 6d), the Guangzhou specimens exhibited a pronounced reduction and the shortest mean fatigue life. In contrast, the Hangzhou specimens showed a higher mean fatigue life than the reference material, while the Suihua result was slightly lower than the reference value. The relatively large error ranges indicate substantial specimen-to-specimen variability, particularly under compression–compression loading. Therefore, unlike the tension–tension results, the compression–compression data did not show a uniform decrease or a consistent regional ranking across the two stress levels.

Compared with the quasi-static properties, the fatigue results revealed a clearer influence of natural exposure. The quasi-static moduli and strengths fluctuated with exposure duration and did not exhibit a consistent monotonic degradation trend. In contrast, the tension–tension fatigue life decreased markedly after 24 months at both selected stress levels for all three exposure locations. This contrast suggests that environmental aging introduced damage that had not yet produced a systematic reduction in quasi-static properties but substantially affected damage accumulation under cyclic tensile loading. The compression–compression response was more strongly affected by specimen variability and loading level, although pronounced fatigue-life reductions were observed for some exposure groups. Overall, the results indicate a more pronounced response to natural exposure in tension–tension fatigue life after 24 months than in the measured quasi-static properties under the investigated conditions.

## 4. Discussion

### 4.1. Evolution and Regional Differences in Natural-Aging Damage

**Figure 7:**
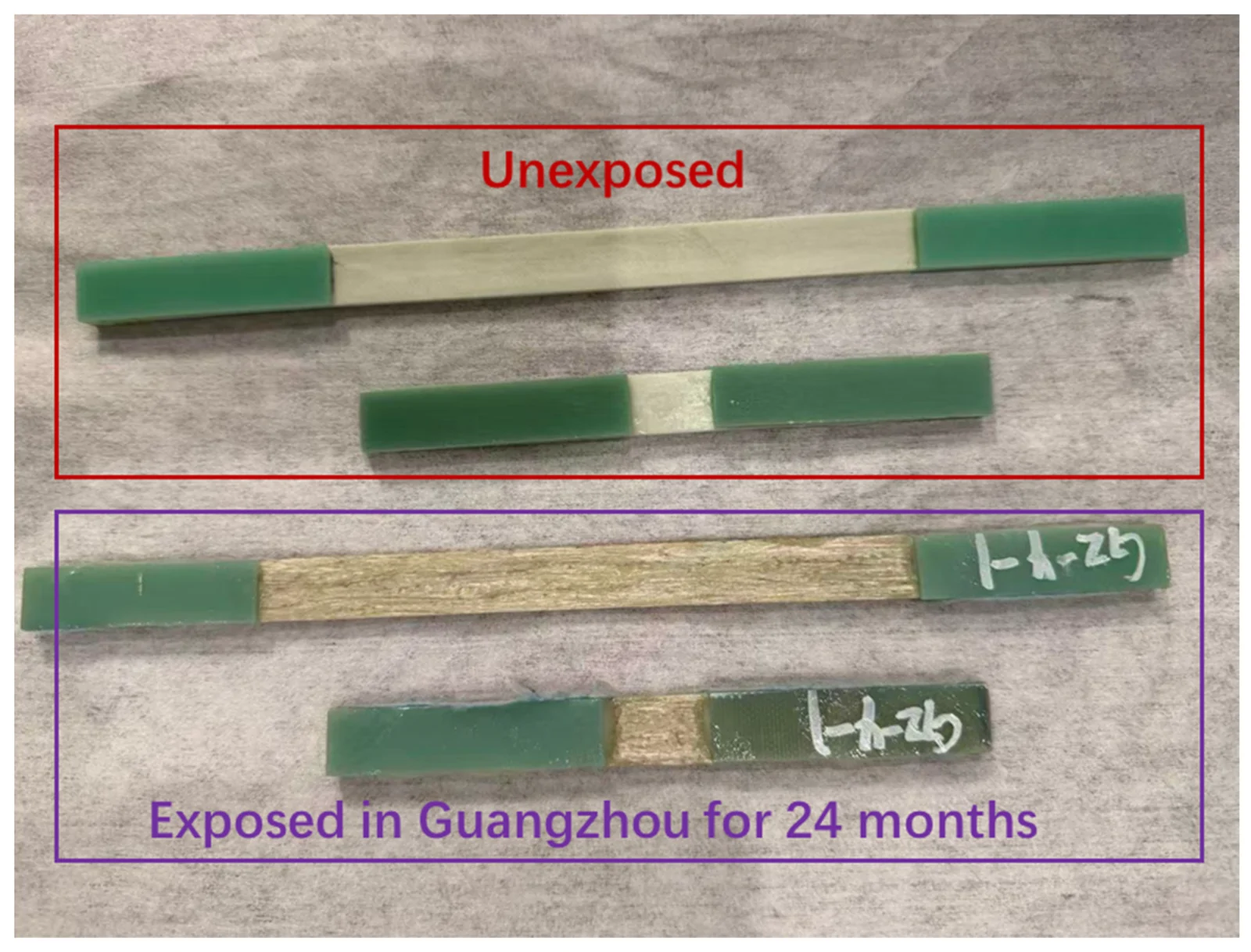
Macroscopic appearance of unexposed GFRP specimens and specimens after 24 months of natural exposure in Guangzhou.

**Figure 8:**
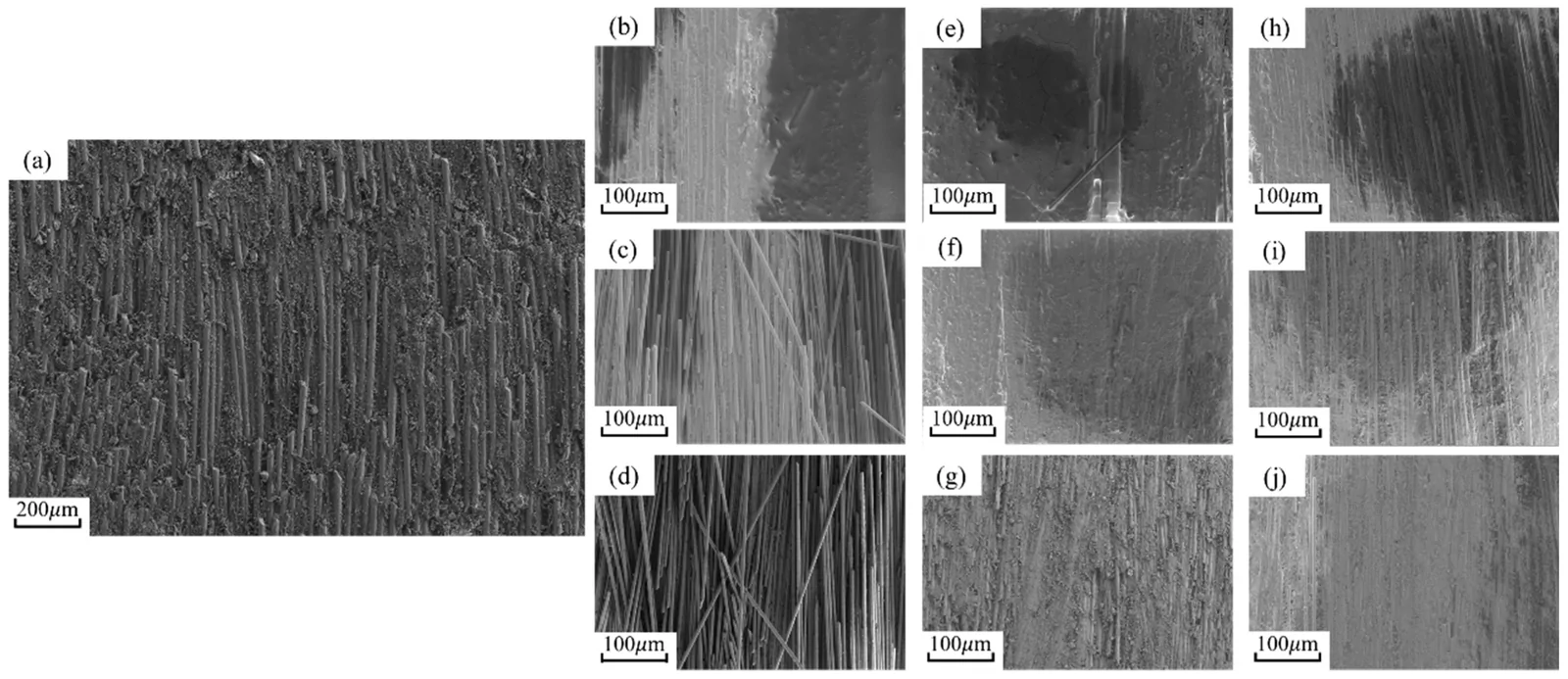
SEM images showing the evolution of the GFRP surface morphology during natural exposure: (**a**) unexposed specimen; specimens exposed in Guangzhou for (**b**) 3, (**c**) 12, and (**d**) 24 months; specimens exposed in Hangzhou for (**e**) 3, (**f**) 12, and (**g**) 24 months; and specimens exposed in Suihua for (**h**) 3, (**i**) 12, and (**j**) 24 months.

Figure 7 compares the macroscopic appearance of the unexposed specimens and specimens exposed in Guangzhou for 24 months. The unexposed specimens exhibited relatively smooth surfaces, whereas the exposed specimens showed a more pronounced fibrous surface texture. These visible changes extended over much of the exposed specimen length rather than being confined to isolated patches. The corresponding microscopic surface features are examined in Figure 8. The SEM observations demonstrate that natural exposure primarily affected the surface resin and the local fiber-matrix region during the investigated 24-month period. As shown in Figure 8a, the unexposed specimen retained relatively continuous resin coverage around the longitudinal fibers. After 3 months of exposure, localized resin fragmentation was already visible in all three regions, although the extent of the damage differed. With increasing exposure duration, resin loss became more extensive and a greater number of fibers were exposed.

The most pronounced morphological evolution was observed in the Guangzhou specimens. At 12 months, large areas of the surface resin had been removed, leaving bundles of exposed fibers. After 24 months, only limited residual resin remained in some regions, and extensive fiber exposure and local fiber separation were evident. The Hangzhou and Suihua specimens also exhibited progressive resin fragmentation and fiber exposure, but the residual matrix coverage was generally greater and fiber separation was less extensive than in the Guangzhou specimen. The differences between Hangzhou and Suihua became less apparent after 24 months.

The surface deterioration observed in the present study reflects exposure to a combination of environmental factors. Studies of other polymer systems under controlled environmental exposure illustrate how the aging response depends on both the material and the exposure conditions. Reyes-Flores et al. [34] reported different mechanical responses to humidity and UV exposure in additively manufactured short-carbon-fiber-reinforced PA6 and used FTIR analysis to investigate the associated molecular changes. Hernández and d’Almeida [35] observed changes in mass, volume, crystallinity, and mechanical response in PA12 aged in oil at different temperatures and pressures, although no significant changes in chemical structure were detected. These findings highlight the importance of considering both physical and chemical changes when interpreting polymer aging. However, differences in matrix chemistry, reinforcement, and exposure conditions limit direct quantitative comparison with the present epoxy-based GFRP.

During natural outdoor exposure, temperature, moisture, precipitation, and solar radiation act together and vary seasonally. The meteorological records in Table 2 characterize the regional exposure conditions, but the more pronounced deterioration of the Guangzhou specimens cannot be assigned to an individual climatic factor because the specimens experienced the combined action of naturally varying environmental conditions. Furthermore, mass change, moisture uptake, glass-transition temperature, and chemical structure were not measured, so the underlying physical and chemical mechanisms could not be identified from the mechanical and SEM observations alone. The present findings show progressive surface resin loss and fiber exposure. After 24 months, the mean tension–tension fatigue life was lower than that of the unexposed material at both selected stress levels for all three sites, whereas the quasi-static properties showed no consistent monotonic trend over the exposure period. The relationship between these morphological observations and the mechanical responses is discussed in the following sections.

### 4.2. Relationship Between Microscopic Damage and Quasi-Static Properties

Although natural exposure caused pronounced surface deterioration, the quasi-static tensile properties did not exhibit a consistent monotonic reduction. This apparent discrepancy can be explained by the spatial distribution and limited extent of the aging damage. As shown in Figure 8, natural exposure primarily resulted in fragmentation and loss of the surface resin, particularly in the Guangzhou specimens. However, the surface layer represented only a small proportion of the specimen cross-section, while most of the internal fibers and matrix remained capable of carrying load. Consequently, extensive surface damage did not necessarily lead to an immediate reduction in the bulk tensile properties.

This effect was particularly evident in the longitudinal tensile response. Because the material contained a high proportion of continuous glass fibers aligned with the loading direction, its longitudinal stiffness and strength were mainly controlled by the intact fibers within the specimen interior. During quasi-static loading, the load could be redistributed from locally damaged surface regions to the undamaged internal fibers. The surface resin loss observed by SEM therefore had a limited effect on the measured longitudinal tensile modulus and strength. This explains why the quasi-static tensile properties fluctuated rather than showing a stable decrease with exposure duration.

**Figure 9:**
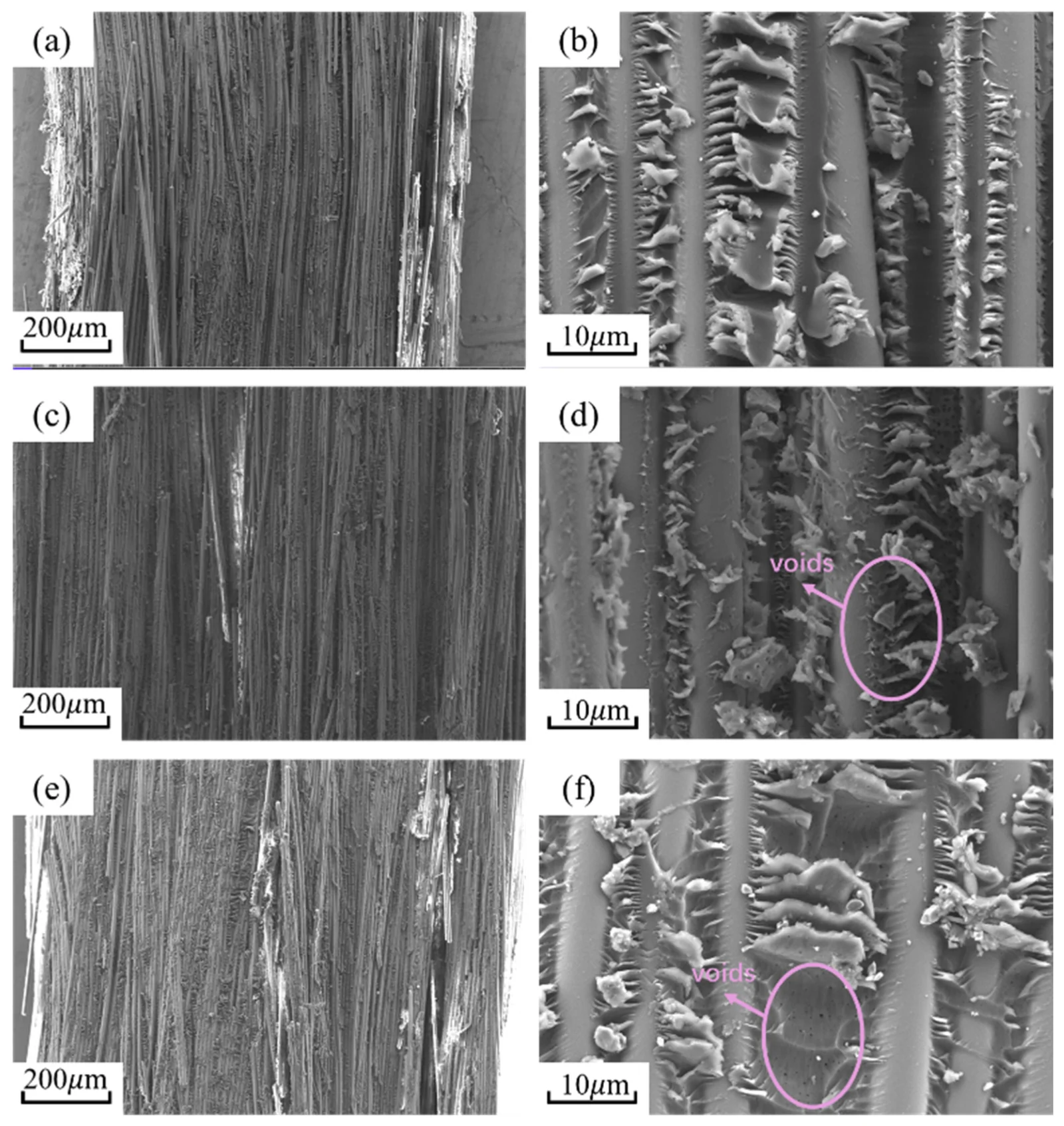
SEM images of the quasi-static fracture surfaces: (**a**,**b**) unexposed specimens and specimens exposed for 24 months in (**c**,**d**) Guangzhou and (**e**,**f**) Hangzhou.

The fracture morphologies in Figure 9 also revealed local features within the specimens. Compared with the unexposed specimen, small void-like regions and local discontinuities were more apparent between the fibers and residual matrix in the specimens exposed for 24 months. These features are consistent with local matrix or interfacial damage, although their origin cannot be determined from the fracture surfaces alone. However, the observed internal defects were small and sparsely distributed, and the surrounding fibers and matrix still formed a largely continuous load-bearing structure. These observations were not accompanied by a consistent reduction in quasi-static strength, although the contribution of the observed defects could not be quantified. The relatively low final strengths of the Guangzhou specimens are broadly consistent with their more severe surface deterioration and more apparent local internal defects. However, because similar reductions were not observed consistently at the preceding exposure stages, these differences cannot be interpreted as a uniform quasi-static degradation trajectory. The results therefore indicate that natural exposure was accompanied by surface deterioration, while the quasi-static properties showed no consistent monotonic decline over the investigated period.

### 4.3. Aging-Induced Fatigue Degradation

In contrast to the quasi-static properties, the tension–tension fatigue life showed a clear reduction after 24 months of natural exposure. At both selected stress levels, all three exposed groups exhibited lower mean fatigue lives than the unexposed reference material, and the Guangzhou specimens consistently showed the lowest mean fatigue life. These results indicate a more pronounced response to natural exposure in tension–tension fatigue life than in the measured quasi-static properties under the investigated conditions. Because fatigue testing of the exposed material was performed only after 24 months, this comparison does not establish when the fatigue-life reduction first emerged during exposure. The reduction in fatigue life may be associated with the progressive accumulation of localized matrix and interfacial damage under cyclic loading. As discussed in Section 4.2, surface resin loss and local discontinuities were observed after natural exposure, while the quasi-static strength showed no consistent monotonic decline. Under cyclic loading, however, local resin cracks, void-like defects, and fiber–matrix discontinuities can act as preferential sites for fatigue-damage initiation. Repeated loading progressively enlarges these defects and reduces the efficiency of stress transfer between neighboring fibers. The viscoelastic response of the epoxy matrix, including molecular rearrangement and relaxation, may also influence stress redistribution during cyclic loading. These effects were not separately quantified in the present study. The fatigue results are therefore interpreted as relative responses following different environmental exposures under the same prescribed cyclic loading conditions, without attributing the observed differences specifically to stress relaxation. The progressive accumulation of local damage may therefore shorten fatigue life even when changes in quasi-static properties remain limited.

**Figure 10:**
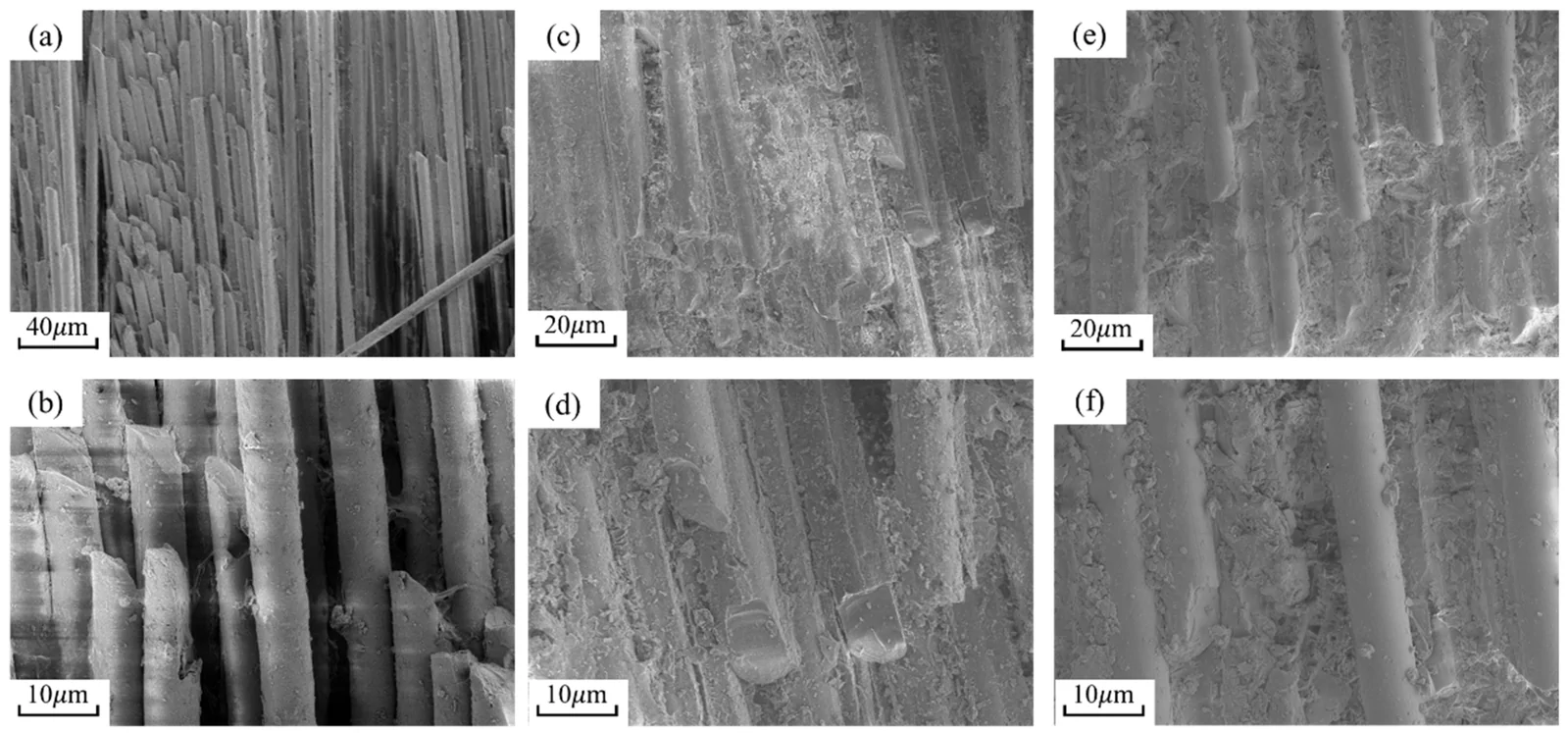
Tensile–tensile fatigue test fracture. (**a**,**b**) Guangzhou, 24 months. (**c**,**d**) Hangzhou, 24 months. (**e**,**f**) Suihua, 24 months.

The tension–tension fatigue fracture morphologies in Figure 10 are consistent with this interpretation. The Guangzhou specimen, shown in Figure 10a,b, exhibited extensive fiber exposure and separation, with relatively little residual resin adhering to many fiber surfaces. The comparatively clean fibers and gaps between adjacent fibers suggest that crack propagation occurred preferentially along locally weakened fiber–matrix regions, resulting in extensive interfacial separation and fiber pull-out. This morphology is consistent with the severe surface resin loss observed in Figure 8 and the comparatively short tension–tension fatigue lives of the Guangzhou specimens.

In comparison, the Hangzhou fracture surface in Figure 10c,d retained more fragmented resin around the fibers and exhibited a rougher fracture morphology. Local fiber breakage and matrix remnants indicate that fatigue-crack propagation involved both matrix fracture and fiber failure rather than proceeding predominantly along the fiber–matrix regions. The Suihua specimen in Figure 10e,f displayed a mixed morphology, including exposed or pulled-out fibers together with regions containing residual matrix. The greater amount of matrix remaining on the Hangzhou and Suihua fracture surfaces suggests that their fiber–matrix load-transfer capability was better preserved than that of the Guangzhou specimen, consistent with their longer mean fatigue lives.

The SEM observations do not, however, provide a definitive explanation for the relative ranking of Hangzhou and Suihua. Hangzhou exhibited the longest mean fatigue life at the lower tensile stress level, whereas Suihua showed the longest life at the higher stress level. This reversal may be related to the heterogeneous distributions of surface damage, internal voids, fiber alignment, and local interfacial defects. Because each SEM image represents only a limited fracture region, small differences between individual images cannot be regarded as representative of the entire specimen.

The compression–compression fatigue results did not show the same consistent reduction and exhibited substantially greater scatter. Compression-dominated fatigue is particularly sensitive to fiber misalignment, initial voids, loading eccentricity, matrix shear damage, and local fiber micro buckling. Small variations in these factors can produce large differences in fatigue life and may obscure the environmental effect. The pronounced scatter observed in Figure 6c,d is consistent with this sensitivity.

## 5. Conclusions

This study investigated the quasi-static properties, fatigue behavior, and microscopic damage of pultruded GFRP after natural exposure in Guangzhou, Hangzhou, and Suihua. The following conclusions can be drawn:

1. During the 24-month natural exposure, the quasi-static properties fluctuated without showing a consistent monotonic degradation trend. In contrast, the tension–tension fatigue lives of all three exposed groups decreased at both selected stress levels, with the Guangzhou specimens exhibiting the shortest mean fatigue life. The compression–compression fatigue results showed substantial scatter and no consistent regional ranking. SEM observations revealed progressive resin loss and fiber exposure, with more pronounced surface deterioration and fiber–matrix separation in the examined regions of the Guangzhou specimens.
2. The combined mechanical and microscopic results suggest that localized matrix and interfacial damage may contribute to reduced fatigue resistance even when quasi-static properties show no consistent monotonic degradation trend. Under the investigated conditions, tension–tension fatigue life provided a more sensitive indicator of the effects of 24-month natural exposure than the measured quasi-static properties. Future work should integrate environmental exposure, matrix and interfacial damage evolution, and mechanical response into a unified predictive framework for assessing residual strength and fatigue life. Such a framework would help quantify the relationships between exposure history, damage accumulation, and long-term performance.

## Figures and Tables

**Table 1:** Natural exposure conditions.

Location	Representative Climate	Duration	Retrieval Intervals for Static Tests
Guangzhou	Warm-humid subtropical	24 months	3, 6, 9, 12, 15, 18, and 24 months
Hangzhou	Subtropical monsoon	24 months	3, 6, 9, 12, 15, 18, and 24 months
Suihua	Cold continental	24 months	3, 6, 9, 12, 15, 18, and 24 months

Note: Fatigue testing of the exposed material was conducted only after 24 months of exposure.

**Table 2:** Monthly meteorological records for the representative stations during the outdoor exposure period (January 2024–December 2025).

Month	Guangzhou: T/RH/P/S	Hangzhou: T/RH/P/S	Suihua: T/RH/P/S
January 2024	14.6/77.4/18.3/131.4	7.0/65.7/38.7/164.6	−18.1/68.0/4.2/173.9
February 2024	15.7/79.2/16.8/86.4	6.9/78.1/239.5/101.9	−12.0/64.3/3.0/208.8
March 2024	19.0/80.4/93.4/65.1	13.7/65.6/60.0/208.2	−2.8/57.7/17.5/235.4
April 2024	24.4/87.0/534.1/46.6	18.6/80.2/217.5/125.5	9.7/50.7/23.3/257.4
May 2024	24.7/85.2/565.7/76.9	22.2/70.3/141.2/242.8	14.8/50.3/73.6/242.3
June 2024	27.9/84.9/339.8/98.0	24.5/84.1/442.9/145.9	19.1/75.1/179.9/193.4
July 2024	29.4/79.6/226.3/183.7	31.9/68.2/38.0/242.4	24.4/75.1/128.9/253.5
August 2024	28.6/84.3/461.3/154.8	31.9/68.2/90.1/248.5	23.0/87.7/188.2/184.5
September 2024	27.8/83.9/140.8/187.2	28.2/77.0/126.6/142.0	15.7/70.3/34.2/215.5
October 2024	24.8/69.7/1.0/239.1	19.5/81.1/123.5/78.3	7.1/62.2/29.6/197.0
November 2024	20.5/73.3/59.7/145.0	15.3/73.1/117.0/126.9	−2.9/68.8/30.8/131.6
December 2024	14.5/64.4/—/208.0	7.6/64.8/—/132.0	−15.4/73.0/—/155.4
January 2025	13.7/62.6/3.4/235.4	7.2/56.7/13.6/195.4	−17.0/76.3/1.2/163.4
February 2025	15.5/71.1/8.7/95.6	6.8/65.2/54.8/117.5	−14.1/67.4/4.8/193.9
March 2025	17.9/77.1/88.5/131.7	12.9/62.0/107.4/156.0	−2.4/54.5/28.4/235.1
April 2025	22.5/76.9/110.5/136.1	20.1/55.5/99.6/213.1	8.9/59.9/31.4/207.5
May 2025	25.3/82.9/509.3/88.8	23.2/64.7/188.6/193.2	15.1/54.4/49.4/247.4
June 2025	27.9/83.2/453.6/119.0	26.1/77.7/347.1/124.6	20.9/72.6/239.4/228.6
July 2025	28.8/82.3/226.5/183.5	30.2/71.2/490.2/189.0	25.1/74.3/62.7/249.7
August 2025	27.3/85.7/569.3/158.2	32.1/61.4/1.6/301.0	21.3/78.9/171.6/212.5
September 2025	27.5/82.8/335.3/188.6	28.6/75.4/158.7/169.7	17.5/70.4/37.8/221.1
October 2025	25.1/77.8/22.6/170.4	23.0/72.7/26.8/109.0	4.5/59.4/14.2/192.8
November 2025	18.9/68.8/8.9/162.6	13.9/64.4/28.5/154.8	−3.0/62.4/10.1/150.1
December 2025	17.2/76.9/26.1/191.9	9.8/62.7/18.6/152.7	−15.1/64.2/10.0/155.6

Notes: T = monthly mean air temperature (°C); RH = monthly mean relative humidity (%); P = monthly total precipitation (mm); S = monthly sunshine duration (h). Data were obtained from the China Meteorological Yearbook query service maintained by the National Meteorological Information Centre, China Meteorological Administration.

## Data Availability

The original contributions presented in this study are included in the article. Further inquiries can be directed to the corresponding author.

## Appendix A

**Table A1:** Mean and standard deviation of transverse tensile properties after natural exposure.

Exposure Duration (Months)	Guangzhou: Modulus (GPa)	Guangzhou: Strength (MPa)	Hangzhou: Modulus (GPa)	Hangzhou: Strength (MPa)	Suihua: Modulus (GPa)	Suihua: Strength (MPa)
3	10.26 ± 1.59	44.11 ± 5.37	10.17 ± 1.33	51.50 ± 0.51	9.75 ± 3.34	42.64 ± 5.35
6	14.64 ± 2.43	42.09 ± 2.00	8.27 ± 2.92	52.78 ± 0.86	6.09 ± 1.60	45.14 ± 2.11
9	11.54 ± 0.71	40.68 ± 1.54	9.23 ± 0.93	45.96 ± 5.48	8.25 ± 1.45	49.00 ± 2.47
12	8.82 ± 0.26	42.13 ± 2.16	10.68 ± 1.13	50.19 ± 3.95	11.80 ± 1.93	43.29 ± 5.32
15	12.53 ± 3.99	45.58 ± 2.05	11.69 ± 1.71	55.44 ± 3.01	9.07 ± 0.21	52.23 ± 1.37
18	8.93 ± 1.49	40.96 ± 1.05	13.00 ± 2.00	50.17 ± 1.04	7.74 ± 0.25	48.11 ± 1.16
24	11.16 ± 0.33	34.63 ± 4.01	12.14 ± 0.55	48.03 ± 5.13	12.18 ± 0.30	48.36 ± 1.05

**Table A2:** Mean and standard deviation of longitudinal tensile properties after natural exposure.

Exposure Duration (Months)	Guangzhou: Modulus (GPa)	Guangzhou: Strength (MPa)	Hangzhou: Modulus (GPa)	Hangzhou: Strength (MPa)	Suihua: Modulus (GPa)	Suihua: Strength (MPa)
3	55.78 ± 0.72	868.98 ± 112.63	56.17 ± 1.21	743.71 ± 13.79	53.29 ± 1.18	683.03 ± 65.25
6	53.54 ± 0.53	720.47 ± 26.18	55.26 ± 2.50	840.14 ± 77.23	54.13 ± 1.87	672.33 ± 138.99
9	56.02 ± 1.43	867.34 ± 36.52	54.09 ± 1.84	764.46 ± 51.28	55.72 ± 1.14	674.91 ± 10.07
12	54.89 ± 1.25	824.76 ± 10.31	53.99 ± 1.24	743.17 ± 91.88	48.81 ± 2.61	639.15 ± 70.70
15	52.71 ± 0.47	788.04 ± 85.04	56.71 ± 2.01	767.11 ± 41.36	54.21 ± 1.06	816.40 ± 6.03
18	50.08 ± 4.17	682.62 ± 174.55	57.00 ± 1.00	796.67 ± 17.24	51.29 ± 1.30	713.52 ± 81.29
24	49.95 ± 2.19	649.19 ± 61.48	54.18 ± 3.90	727.64 ± 67.48	53.55 ± 2.22	777.76 ± 140.96

**Table A3:** Mean and standard deviation of longitudinal compressive properties after natural exposure.

Exposure Duration (Months)	Guangzhou: Modulus (GPa)	Guangzhou: Strength (MPa)	Hangzhou: Modulus (GPa)	Hangzhou: Strength (MPa)	Suihua: Modulus (GPa)	Suihua: Strength (MPa)
3	43.07 ± 2.47	577.00 ± 89.56	41.18 ± 0.81	581.47 ± 16.88	48.04 ± 0.43	458.69 ± 43.14
6	48.53 ± 3.42	441.39 ± 16.93	39.09 ± 0.93	601.03 ± 47.03	45.77 ± 0.69	448.23 ± 18.20
9	47.93 ± 6.25	588.44 ± 152.32	47.34 ± 0.75	657.03 ± 19.21	55.05 ± 10.52	467.99 ± 193.59
12	63.91 ± 8.31	393.11 ± 60.49	45.05 ± 2.42	621.68 ± 75.01	41.90 ± 1.85	479.55 ± 63.42
15	43.99 ± 5.33	471.65 ± 79.46	45.80 ± 3.06	673.94 ± 6.92	54.29 ± 7.31	408.36 ± 27.19
18	52.96 ± 4.40	464.92 ± 26.64	47.00 ± 1.40	685.88 ± 23.43	41.83 ± 3.65	415.96 ± 5.16
24	44.72 ± 5.18	398.91 ± 39.41	51.98 ± 14.75	664.07 ± 59.84	57.68 ± 10.19	439.20 ± 65.98
